# FRAMEWORK-BASED EVALUATION OF MOBILE HEALTH APPS FOR OLDER ADULTS: IMPLICATIONS FOR TECHNOLOGY-ENABLED SELF-CARE

**DOI:** 10.1093/geroni/igae098.2900

**Published:** 2024-12-31

**Authors:** Priya Nambisan, Akshat Kapoor, Gary Kreps, Reshma Sahithi Dangeti

**Affiliations:** University of Wisconsin Milwaukee, Milwaukee, Wisconsin, United States; East Carolina University, Greenville, North Carolina, United States; George Mason University, Fairfax, Virginia, United States; University of Wisconsin Milwaukee, Milwaukee, Wisconsin, United States

## Abstract

The rapidly growing older adult population which is burdened with multiple chronic conditions (MCC) and declining physical and mental functionality, portends opportunities for developing better technologies to cater to their self-care support needs. However, it is not clear whether the current set of health apps targeted at older adults are suitable, or whether they fulfill their health and self-care support needs of older adults with MCC. We conducted a systematic review using the PRISMA method and also used a theoretical framework to evaluate currently available health apps for older adults. Many studies that review apps merely review their features, however, to get a more in-depth and nuanced understanding of whether the apps are meeting the needs of older adults, we utilize a needs-based theoretical framework. This framework was specifically developed to assess the needs of this population group, and the technological elements that could be leveraged for meeting their health needs. The framework delineates seven needs of older adults in the context of technology applications: 1) prevention focus; 2) comprehensiveness; 3) education and training for self-care; 4) community and home-based care; 5) social and emotional support; 6) privacy and security; and 7) positive experiences. Our results indicate that many apps in the market do not fulfill the needs of older adults with MCC. Our systematic review did not yield even a single comprehensive app for older adults with MCC which covered all the needs comprising the framework, thus highlighting the digital exclusion of this population and their needs for support.

